# *NNT* Pseudoexon Activation as a Novel Mechanism for Disease in Two Siblings With Familial Glucocorticoid Deficiency

**DOI:** 10.1210/jc.2014-3641

**Published:** 2014-12-02

**Authors:** Tatiana V. Novoselova, Shoshana R. Rath, Karen Carpenter, Nicholas Pachter, Jan E. Dickinson, Glynis Price, Li F. Chan, Catherine S. Choong, Louise A. Metherell

**Affiliations:** Centre for Endocrinology (T.V.N., L.F.C., L.A.M.), William Harvey Research Institute, John Vane Science Centre, Queen Mary, University of London, London, EC1M 6BQ, United Kingdom; Department of Endocrinology (S.R.R., G.P., C.S.C.), Princess Margaret Hospital, Child and Adolescent Services, Subiaco, Perth, Western Australia 6008; Department of Diagnostic Genomics (K.C.), PathWest Laboratory Medicine, Nedlands, Western Australia 6009; Genetic Services of Western Australia (N.P.), King Edward Memorial Hospital, Subiaco, Western Australia 6008; and School of Pediatrics and Child Health (S.R.R., N.P., C.S.C.), and School of Women's and Infants' Health (J.E.D.), University of Western Australia, Perth, Australia 6009

## Abstract

**Context:**

Intronic DNA frequently encodes potential exonic sequences called pseudoexons. In recent years, mutations resulting in aberrant pseudoexon inclusion have been increasingly recognized to cause disease.

**Objectives:**

To find the genetic cause of familial glucocorticoid deficiency (FGD) in two siblings.

**Patients:**

The proband and his affected sibling, from nonconsanguineous parents of East Asian and South African origin, were diagnosed with FGD at the ages of 21 and 8 months, respectively.

**Design:**

Whole exome sequencing was performed on genomic DNA (gDNA) of the siblings. Variants in genes known to cause FGD were assessed for causality. Further analysis of gDNA and cDNA was performed by PCR/RT-PCR followed by automated Sanger sequencing.

**Results:**

Whole exome sequencing identified a single, novel heterozygous variant (p.Arg71*) in nicotinamide nucleotide transhydrogenase (NNT) in both affected individuals. Follow-up cDNA analysis in the proband identified a 69-bp pseudoexon inclusion event, and Sanger sequencing of his gDNA identified a 4-bp duplication responsible for its activation. The variants segregated with the disease: p.Arg71* was inherited from the mother, the pseudoexon change was inherited from the father, and an unaffected sibling had inherited only the p.Arg71* variant.

**Conclusions:**

FGD in these siblings is caused by compound heterozygous mutations in *NNT*; one causing pseudoexon inclusion in combination with another leading to Arg71*. Discovery of this pseudoexon activation mutation highlights the importance of identifying sequence changes in introns by cDNA analysis. The clinical implications of these findings include: facilitation of antenatal genetic diagnosis, early institution of potentially lifesaving therapy, and the possibility of preventative or curative intervention.

Nicotinamide nucleotide transhydrogenase (*NNT*) is a highly conserved gene, coding for a mitochondrial protein that protects cells from oxidative stress. Mutations in *NNT* may result in increased rates of cellular apoptosis and therefore dysfunction of the affected tissue. Although this protein is ubiquitously expressed in human tissues, mutations are to date associated primarily with adrenal insufficiency (1) and have previously been associated with familial glucocorticoid deficiency (FGD). Some mouse models have detected impaired insulin secretion and glucose intolerance due to oxidative stress on pancreatic B cells (2), but this has not been described in humans. FGD is characterized by isolated glucocorticoid deficiency due to the failure of the adrenal cortex to respond to ACTH (reviewed in Ref. 3). Patients with FGD generally present with Addisonian symptoms, including hyperpigmentation of skin and mucous membranes, with the important distinction that mineralocorticoid production is preserved (3). FGD has been associated with mutations in seven genes: *MC2R*, *MRAP*, *STAR*, *CYP11A1*, *NNT*, *MCM4*, and *TXNRD2* (3, 4). Most commonly (50% of cases), *MC2R* and *MRAP* defects are responsible.

To identify and join exons (the DNA sequences that code for proteins), the splicing machinery must recognize and differentiate noncoding introns from exons and exclude them from pre-mRNA (5). Aberrant splicing events are commonly recognized in disease states, but these usually occur when the consensus sequences flanking exons are altered (6). Introns often contain sequences similar to exons with canonical 5′ and 3′ splice sites surrounded by typical flanking regions (7). However, these are ignored by the cellular splicing machinery and not incorporated into mature mRNA. They are therefore termed pseudoexons (8). In some genes, like human *HPRT*, pseudoexons outnumber exons by as many as 10:1 (9). This phenomenon has mostly been studied in relation to splicing regulation mechanisms, but recently the importance of pseudoexons has been re-evaluated as mutations resulting in aberrant pseudoexon inclusion have been found to be disease causing in more than 50 genes (10). Most pathological pseudoexon inclusion events originate from the creation of a new donor or acceptor splice site within an intronic sequence, but some alter splice regulatory elements as previously described by our group in the GH receptor (11). Here we demonstrate that compound heterozygosity for a pseudoexon activation event, together with a stop-gain mutation in *NNT*, results in FGD in two siblings.

## Patients and Methods

**Figure 1.**
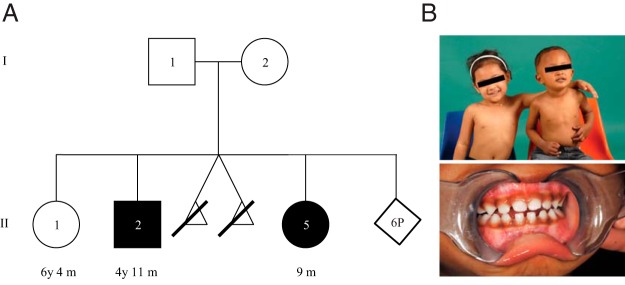
A, Pedigree of FGD family, filled symbols representing affected individuals. B, Upper panel, proband II:2 (on the right) with his unaffected sibling II:1. Lower panel, note the increased skin and gum pigmentation of the proband.

The proband (Figure 1A, II:2) was born by normal vaginal delivery after an uneventful pregnancy with a birth weight of 3130 g. He had an unremarkable first year of life, attaining developmental milestones appropriately, although his mother commented retrospectively that she had noted increasing pigmentation from about 6 months of age. At 12 months of age, after a gastrointestinal illness, he was found comatose by his father. Upon presentation, venous blood glucose level was 0.6 mmol/L (normal range [NR], 3.0–5.4 mmol/L), cortisol level was 560 nmol/L (NR, 138–635 nmol/L), sodium was low at 125 mmol/L (NR, 134–146 mmol/L), and potassium was normal at 4.7 mmol/L (NR, 3.4–5.0 mmol/L). A presumptive diagnosis of ketotic hypoglycemia secondary to gastroenteritis was made. At 21 months of age, subsequent to a brief history of viral upper respiratory tract symptoms, the proband was once again found unresponsive with hypoglycemia (serum blood glucose, 1.4 mmol/L). Endocrine evaluation indicated adrenal insufficiency with low cortisol, 30 nmol/L; elevated plasma ACTH, 492 pmol/L (NR, 2.2–13.3 pmol/L); slightly low serum aldosterone, 192 pmol/L (NR, 300–500 pmol/L for age 1 wk to 2 y); suppressed renin, 2.2 mU/L (NR, 5–100 mU/L for age 1 wk to 2 y), and appropriately suppressed insulin, <2 mU/L. The child had hyperpigmentation of the skin and gums (Figure 1B). Glucocorticoid deficiency was diagnosed, and hydrocortisone therapy commenced.

A sibling (Figure 1A, II:5) 4 years younger than the proband was born at 39 week gestation with birth weight of 2595 g (third percentile). An amniocentesis for increased risk combined first trimester screen (secondary to a pregnancy associated plasma protein A [PAPP-A] < first percentile at 0.160 Multiple of the Median) had returned a normal female karyotype. Serial ultrasound assessments had shown all fetal biometric parameters to be maintained on the fifth percentile with normal fetoplacental Doppler studies. A short Synacthen test was performed on day 3 of life, and 125 μg of Synacthen was administered: baseline cortisol, 38 nmol/L; 30-minute level, 480 nmol/L; and peak attained at 60 minutes, 580 nmol/L (NR, >550 nmol/L). ACTH level was in the normal range at 7.4 pmol/L (NR, 2.0–10 pmol/L), and there was no hypoglycemia on capillary blood glucose monitoring over the first 3 days of life. The infant was clinically well and remained under surveillance. Increased pigmentation was noted by her parents from 4 months of age. At 8 months of age, a second short Synacthen test was undertaken (same dose of Synacthen utilized); peak cortisol level of 130 nmol/L was attained at 60 minutes, consistent with adrenal insufficiency. ACTH at this time was 517 pmol/L (NR, 2.0–10 pmol/L). Aldosterone and renin were in the normal range. Hydrocortisone therapy was commenced immediately. Fludrocortisone was not required for either child.

Both siblings (currently ages 7 and 3 y) have normal thyroid function, glycated hemoglobin, and fasting glucose and insulin levels. Their growth is appropriate, with the proband tracking along the 50th centile for height and 25th for weight and his sibling tracking between the 10th and 50th centiles for height and weight, in line with their midparental expectation (25th centile).

The mother is of East Asian origin, and the father is South African. They are nonconsanguineous, with no family history of adrenal insufficiency. In addition to the two affected siblings, there is a third, unaffected sister 2 years older than the proband (Figure 1A, II:1). The mother is currently pregnant and had an amniocentesis (again for increased risk in the combined first trimester screen). DNA testing of cultured amniocytes excluded FGD in the fetus or heterozygous carriage of either mutation described below. There was also a twin pregnancy that failed early in gestation (Figure 1A). There is no history of unexplained stillbirths or death in childhood on either side of the extended family.

### Study approval

This study was approved by the Outer North East London Research Ethics Committee (reference no. 09/H0701/12). Informed consent was obtained from affected individuals and their family members.

### Mutation discovery

Genomic DNA was extracted from peripheral blood leukocytes of affected individuals and family members after obtaining informed consent from them and/or their parents. Sequencing of coding exon/intron boundaries of the melanocortin 2 receptor (*MC2R*) and the MC2R accessory protein (*MRAP*) had been undertaken, and no mutations were found (primer sequences are provided in Supplemental Table 1).

Whole exome sequencing was conducted on the proband and his affected sister (Otogenetics Corp). The captured libraries were sequenced, and downstream analysis was conducted via DNAnexus (https://dnanexus.com/). Single nucleotide polymorphisms, with threshold coverage of at least 10 reads on the respective nucleotide, were included in the analysis. Variants in the genes associated with FGD (OMIM no. 202200) or nonclassical congenital lipoid adrenal hyperplasia (NC-CLAH) were assessed for causality (*MC2R*, *MRAP*, *NNT*, *MCM4*, and *TXNRD2* for FGD; *STAR* and *CYP11A1* for NC-CLAH). Mutations in *STAR* and *CYP11A1* usually result in CLAH (OMIM no. 201710), a severe disorder with both adrenal and gonadal steroid insufficiencies; however, certain partial loss-of-function mutations can give rise to a milder phenotype with no gonadal derangement, termed NC-CLAH (12). The identified sequence changes in *NNT* were confirmed by PCR, followed by automated sequencing using primers designed to cover the affected regions (primer sequences in Supplemental Table 1).

### cDNA analysis

RNA was extracted using QIAGEN RNeasy Mini Kit (QIAGEN) from phytohaemagglutinin-stimulated blood cells. Cells were prepared using a modification of a technique first reported by Nowell and Hungerford (13). cDNA was prepared from RNA using the Superscript III Kit by Invitrogen. DNA was prepared from EDTA blood using a QIAmp DNA Mini Kit (QIAGEN).

### Reference sequence

*NNT* reference sequences are Ensembl, ENSG00000112992 and ENST00000264663. NCBI reference sequences are NG_032869.1 and NM_012343.3.

## Results

**Figure 2.**
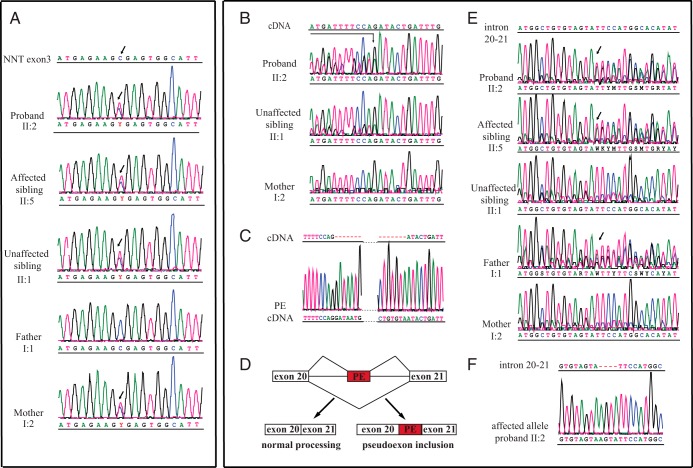
A, The heterozygous sequence change in exon 3 of *NNT* g.[5:43613069C>T] c.[211C>T] p.[Arg71*] inherited from the mother is present in all the children (indicated with arrow). B, The heterozygous sequence change in the cDNA of the proband II:2 (indicated with arrow) is not seen in unaffected sibling II:1 or the mother I:2. C, Partial sequence chromatograms showing the junctions between exon 20/PE (left) and PE/exon 21 (right). The full sequence is shown in Supplemental Figure 2. D, Schematic of the pseudoexon inclusion event into the mRNA. E, The heterozygous sequence change in intron 20–21 (indicated with arrows) showing that the mutation is inherited from the father and is present in both affected siblings, but not the mother or unaffected sister. F, Partial sequence chromatogram of a cloned PCR fragment from the affected allele of proband II:2 containing the *NNT* intron 20–21 AGTA duplication g.[5:43701537_43701540dupAGTA]. PE, pseudoexon.

Whole exome sequencing identified eight variants in FGD-causing genes in the proband and his sister: two in *NNT*, one in *MCM4*, and five in *TXNRD2* (Supplemental Table 2). Variants in the other four genes (*MC2R*, *MRAP*, *STAR*, and *CYP11A1*) were not identified. Four of the identified variants represented synonymous changes (rs10057103, rs1139795, rs35544159, and rs5748470), and three had a minor allele frequency > 10% (rs762679, rs1139793, and rs5748469). The final change was a single, novel, heterozygous variant, g.5:43613069C>T; c. 211C>T; p. Arg71* in exon 3 of the antioxidant defense gene *NNT* (Figure 2A). This heterozygous p.Arg71* variant will result in premature truncation and remove all functional domains of the protein. It was found in both affected individuals (II:2 and II:5) and also in the DNA of the unaffected sibling (II:1) and their mother (I:2) (Figure 2A). The pattern of inheritance of FGD is autosomal recessive, so it was unlikely that, on its own, this heterozygous change was causative—especially because it was also carried by the unaffected mother and sister.

To determine whether there was an intronic lesion on the proband's other allele, cDNA sequencing was undertaken after inhibition of nonsense-mediated mRNA decay (NMD) with cycloheximide (13). This revealed the inclusion of a 69-bp pseudoexonic sequence from the middle of intron 20 of the *NNT* gene: c.2995_2996insNG_032869.1:g.103679_103747; p.Asp999Glyfs*2 (Figure 2, B and C, and Supplemental Figure 1). If translated, this would result in a frameshift and creation of a premature stop codon at position 1000 (p.Asp999Glyfs*2), and therefore truncation of the protein before the NADPH binding domain essential for NNT functionality. No such inclusion was seen in the cDNA of the unaffected sibling (II:1) or their mother (I:2) (Figure 2B).

Further analysis of the genomic DNA of the patient identified a 4-bp duplication, g.[5:43701537_43701540 dupAGTA], within intron 20 (Figure 2, D–F, and Supplemental Figures 1 and 2). No other sequence changes were detected within or between exons 20 and 21. Without the duplication, no splice site is predicted, but splice prediction software predicts the creation of an aberrant donor splice site with duplication of the AGTA. Both Human Splice Finder (http://www.umd.be/HSF/) and Berkeley Drosophila Genome Project splice site prediction (http://www.fruitfly.org/seq_tools/splice.html) software gives the same result. This change was novel, also identified in the affected sibling (II:5), and was inherited from the father (I:1), who does not have the Arg71* variant or FGD (Figures 1A and 2E). Neither of the heterozygous *NNT* sequence changes identified in this family have been annotated in dbSNP or the NHLBI exome variant server (www.ncbi.nlm.nih.gov/SNP/; http://evs.gs.washington.edu/EVS/), and both are predicted to be protein damaging.

## Discussion

The patients reported in this study had a progressive presentation of their FGD, typical for mutations in genes involved in oxidative stress regulation (1, 4).

The genetic analysis demonstrated that in this case FGD was caused by a heterozygous intron 20 mutation in combination with a heterozygous stop-gain mutation in exon 3 of the oxidative defense gene *NNT*. Exome sequencing identified the c.311C>T change but failed to identify the intronic AGTA duplication because exome data usually only covers 50 bp either side of an exon, and the duplication was at a distance of 1197 bp from exon 20. Without the pointer of the first c.311C>T mutation in *NNT*, we would not have performed cDNA analysis to uncover the pseudoexon event. The underlying genomic mutation was detected by conventional Sanger sequencing of intron 20. The duplication in intron 20 resulted in formation of an aberrant splice site, pseudoexon activation, and its inclusion into the cDNA. Pre-mRNA splicing is a complicated and incompletely understood mechanism mediated by degenerative splicing elements consisting of the branch point sequence, the polypyrimidine tract, the 5′ (donor) and 3′ (acceptor) splice site, and exonic/intronic splicing enhancers/silencers. Within the 5′ and 3′ splice sites, the GT and AG nucleotides, respectively, are almost invariant, but the combination of nucleotides flanking them is also essential for recognition by the splicing machinery. In this case, the duplication AGTAAGTA renders the first GT a 5′ splice site, whereas in the single AGTA, the GT is not a valid splice site. A limitation of this study is that we were unable to perform a Western blot to confirm the absence of the protein because we could not obtain further samples from the family.

This case highlights the importance of cDNA analysis for detection of significant mutations in the intronic sequence of candidate genes in instances where exon sequencing fails to provide an adequate diagnosis. Intronic sequences are often excluded from genetic studies due to their large size, coupled with the uncertainty of predicting the consequence of any changes found within them. cDNA analysis offers the ideal alternative, although it may be difficult to perform for tissue-specific genes. Even in ubiquitously expressed genes, cDNA changes could be missed if they result in NMD (14), a process that eliminates mRNAs containing premature termination codons, thus helping to limit the synthesis of abnormal proteins (14). Reducing NMD with cycloheximide treatment, as performed here, facilitates the detection of abnormal transcripts that would otherwise degrade (14). Both of these novel mutations result in premature stop codons, and their mRNA would likely undergo NMD, resulting in the absence of an *NNT* transcript, therefore precluding discovery of the aberrant pseudoexon inclusion. We suspected the presence of an intronic defect because of the clue of one defective *NNT* allele, but should a defect occur in homozygosity, it could easily be missed completely by conventional exonic or whole exome sequencing.

Sequence changes within introns can provide a potential target for antisense oligonucleotide therapy (15), and therefore detection of pseudoexon mutations is becoming increasingly important. If used in these siblings, such oligonucleotides could interfere with the pseudoexon activation sequence and thus block inclusion into the cDNA (Figure 2C), leaving only the heterozygous Arg71* mutation in exon 3, which on its own does not cause FGD (as demonstrated in the unaffected sibling [II:1] and the mother [I:2]) (Figure 1A).

Discovery of the causative mutations in this family has facilitated prenatal testing and exclusion of FGD in the mother's current pregnancy. As demonstrated in this case, complete genetic diagnosis enables antenatal diagnosis, obviating unnecessary investigation for unaffected offspring, while facilitating early initiation of potentially lifesaving therapy in affected offspring. Furthermore, it raises the possibility of preventative or curative intervention in the future.
